# Author Correction: Glucose homeostasis is regulated by pancreatic β-cell cilia via endosomal EphA-processing

**DOI:** 10.1038/s41467-021-24865-4

**Published:** 2021-08-03

**Authors:** Francesco Volta, M. Julia Scerbo, Anett Seelig, Robert Wagner, Nils O’Brien, Felicia Gerst, Andreas Fritsche, Hans-Ulrich Häring, Anja Zeigerer, Susanne Ullrich, Jantje M. Gerdes

**Affiliations:** 1 Institute for Diabetes and Regeneration Research, Helmholtz Zentrum München, Ingolstädter Landstr. 1, 85764 Neuherberg, Germany; 2 Technical University of Munich, Medical Faculty, 81675 Munich, Germany; 3 Department of Internal Medicine IV, Division of Endocrinology, Diabetology, Nephrology, Vascular Disease and Clinical Chemistry, University Hospital of Tübingen, Tübingen, Germany; 4 Institute for Diabetes Research and Metabolic Diseases of the Helmholtz Centre Munich at the University of Tübingen (IDM), Tübingen, Germany; 5 Deutsches Zentrum für Diabetesforschung, DZD, Munich, Germany; 6 Institute for Diabetes and Cancer, Helmholtz Zentrum München, Munich, Germany

**Keywords:** Cell biology, Molecular biology, Physiology, Diseases, Endocrinology

Correction to: *Nature Communications* 10.1038/s41467-019-12953-5, published online 12 December 2019.

In the original version of this article the Figure Legends in the Supplementary Information were inadvertently omitted. The HTML of the article has been updated to include the correct version of the Supplementary Information. The original version of the article also incorrectly referenced Supplementary Figures 2D and Supplementary Figure 2E, which were cited as Figure 2D and Figure 2E. This has been corrected in both the PDF and HTML versions of the Article.

## Supplementary information

Supplemental information

