# Supplementary material for: Glucose homeostasis is regulated by pancreatic β-cell cilia via endosomal EphA-processing
Source: Nat Commun. 2019 Dec 12;10:5686. doi: 10.1038/s41467-019-12953-5 (PMC6908661; doi:10.1038/s41467-019-12953-5)

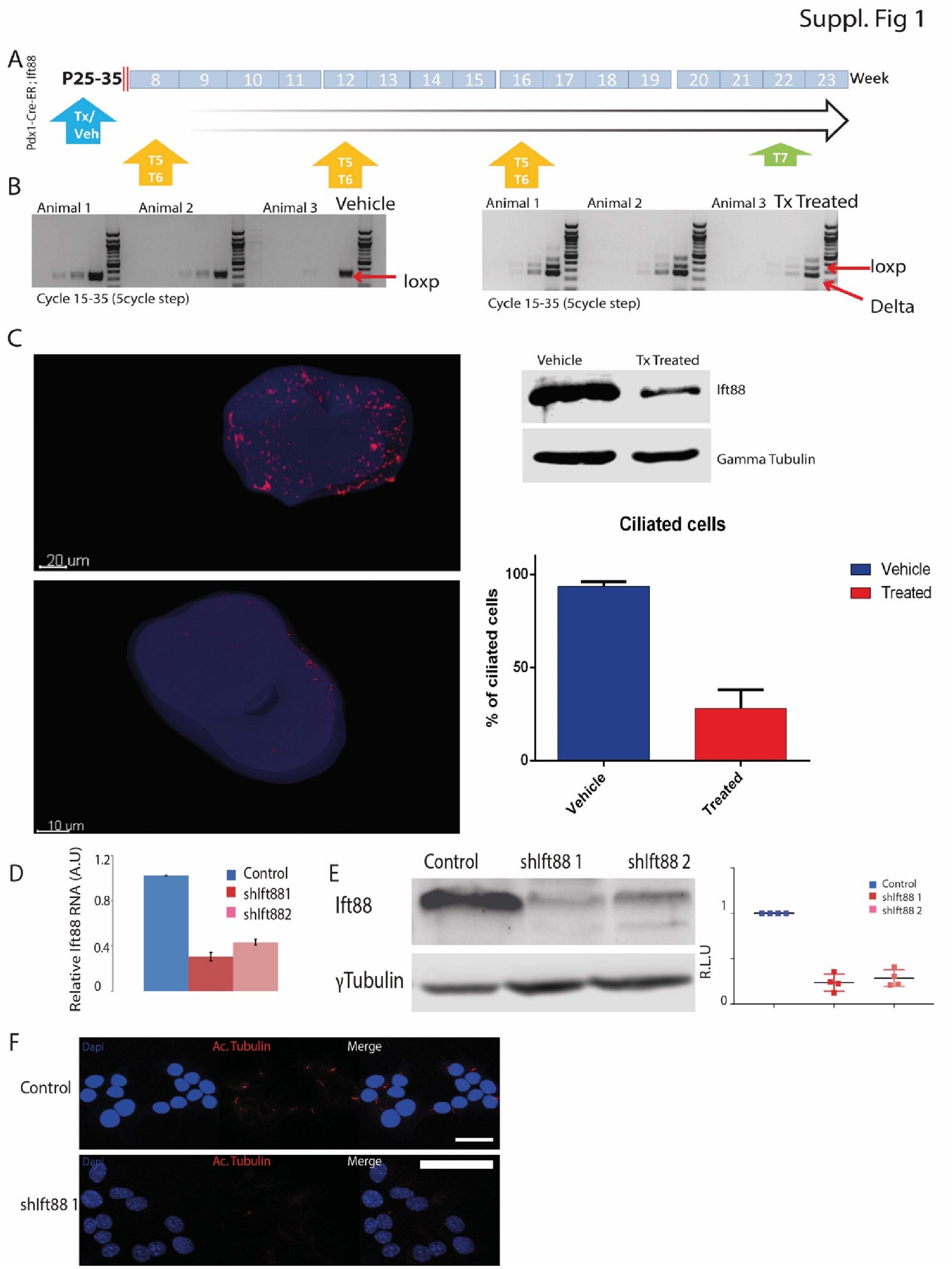
Suppl. Fig 1
A
Pdx1-Cre-ER ; Ift88
P25-35
8 9 10 11 12 13 14 15 16 17 18 19 20 21 22 23 Week
Tx/ Veh
T5 T6
T5 T6
T5 T6
T7
B
Animal 1 Animal 2 Animal 3 Vehicle
loxp
Cycle 15-35 (5cycle step)
Animal 1 Animal 2 Animal 3 Tx Treated
loxp
Delta
Cycle 15-35 (5cycle step)
C
20 um
10 um
Vehicle Tx Treated
Ift88
Gamma Tubulin
Ciliated cells
% of ciliated cells
100
50
0
Vehicle
Treated
Vehicle
Treated
D
Relative Ift88 RNA (A.U)
1.2
0.8
0.4
0
Control
shIft881
shIft882
E
Control shIft88 1 shIft88 2
Ift88
γTubulin
R.L.U
1
0
Control
shIft88 1
shIft88 2
F
Dapi Ac.Tubulin Merge
Control
Dapi Ac.Tubulin Merge
shIft88 1

Supplemental Figure 1: Validation of the βICKO model

A. Schematic timeline of animal experiments. GTT- intraperitoneal Glucose Tolerance test, IST- insulin secretion test, ITT- intraperitoneal insulin secretion test **B.** Semi-quantitative, limited cycle PCR on genomic DNA isolated from primary islets 4 weeks post induction. (100bp size marker). **C.** Primary cilia (red) in isolated islets 4 weeks post induction (Maximum projection, islet outlined in white, dashed line. On the right, quantification of Ift88 protein levels after ex vivo (top panels) and in vivo induction of recombination, mean±s.d.. **D**. mRNA level of Ift88 in MIN6m9 cells, mean±s.d.. **E**. Immunoblot of Ift88 and γ-tubulin in shItf88 expressing MIN6m9 cells and quantification (n=4) , mean±s.d.. **F**. Primary cilia in shIft88 expressing MIN6 m9 cells (acetylated α-tubulin shown in red; scale bar 20µm).

Suppl Fig 2

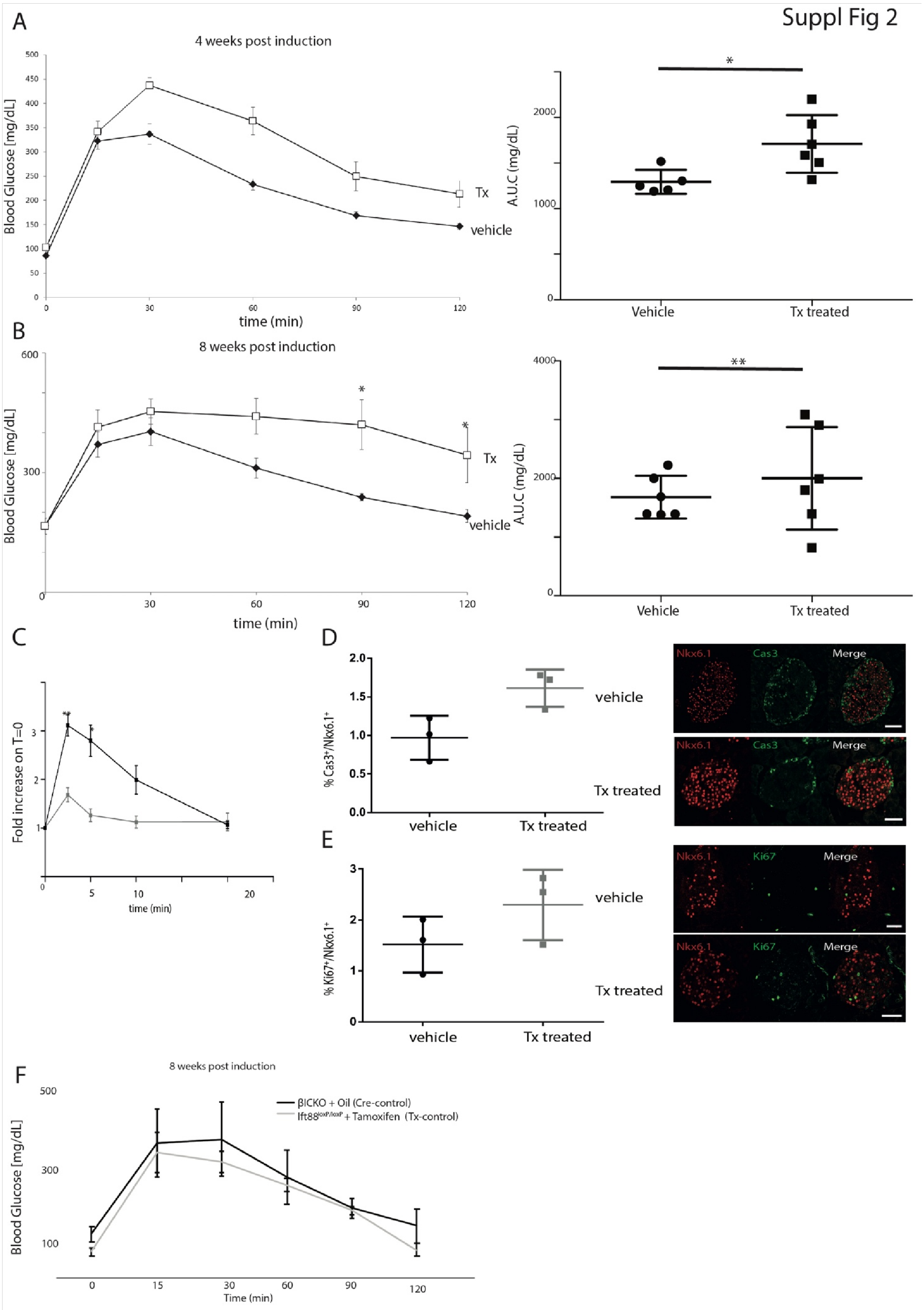
A
4 weeks post induction
Blood Glucose [mg/dL]
time (min)
Tx
vehicle
A.U.C (mg/dL)
Vehicle
Tx treated
B
8 weeks post induction
Blood Glucose [mg/dL]
time (min)
Tx
vehicle
A.U.C (mg/dL)
Vehicle
Tx treated
C
Fold increase on T=0
time (min)
D
% Cas3+/Nkx6.1+
vehicle
Tx treated
Nkx6.1
Cas3
Merge
E
% Ki67+/Nkx6.1+
vehicle
Tx treated
Nkx6.1
Ki67
Merge
F
8 weeks post induction
Blood Glucose [mg/dL]
βICKO + Oil (Cre-control)
Ift88loxP/loxP + Tamoxifen (Tx-control)
Time (min)

Supplemental Figure 2: Progression of the βICKO phenotype over time

A. Intraperitoneal Glucose Tolerance Test 4 week post induction.(n=6, $p<0.0001$ (GTT, Repeated measures one-way ANOVA); p=0.0045 (A.U.C., one way ANOVA, Holm Sidak multiple comparison), mean$\pm$s.e.m. **B**. Intraperitoneal Glucose Tolerance Test 8 week post induction.(n=6, p=0.0013 (GTT, Repeated measures one-way ANOVA); p= 0.005 (A.U.C., one way ANOVA, Holm Sidak multiple comparison) mean$\pm$s.e.m. **C**. Insulin secretion test 8 weeks post induction. (n=3, Group comparison $p<0.0001$ (Repeated measures one-way ANOVA) , mean$\pm$s.e.m). **D**. Percentage of apoptotic beta cells over total of beta cells. Representative images of control and treated animal islets. Nkx6.1 shown in red and caspase-3 in green (n=3 animals, n=7 islet per animal, 6 weeks post induction, scale bar 50µm) , mean$\pm$s.d.. **E**. Percentage of proliferative beta cells over total of beta cells. Representative images of control and Tx-treated animal islets. Nkx6.1 shown in red and Ki67 shown in green (n=3 animals, n=7 islet per animal, 6 weeks induction, scale bar 50µm) , mean$\pm$s.d.. **F**. Intraperitoneal Glucose Tolerance Test eight week post-induction of βICKO mice treated with oil (Cre-Control, n=6) and Ift88$^{loxP/loxP}$ mice treated with Tamoxifen (Tx-Control, n=3, $p>0.05$ (Repeated measures one-way ANOVA) , mean$\pm$s.e.m.).

Suppl Fig 3

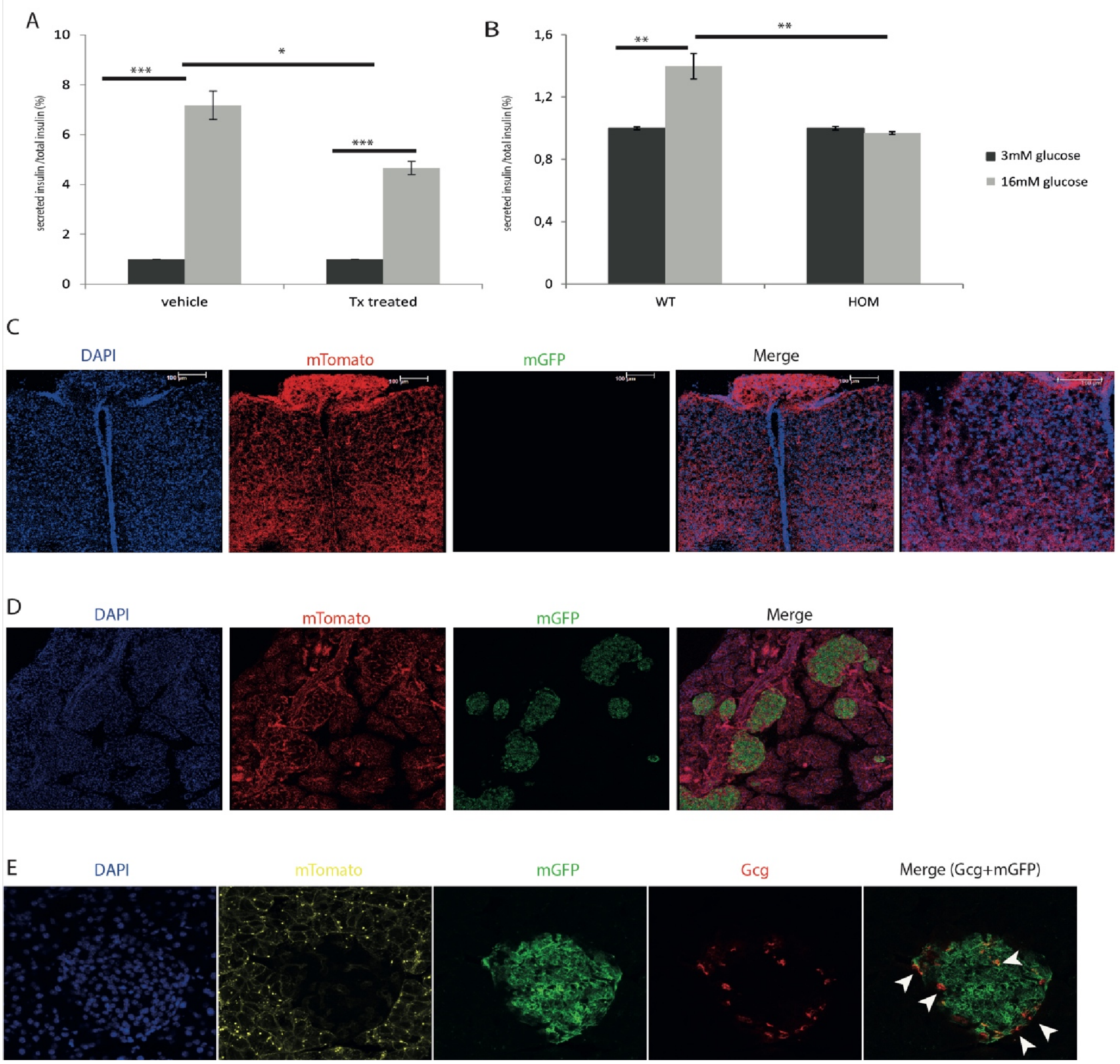

Supplemental Figure 3: PDX1-Cre deletion is specific to β-cells

A. Glucose stimulated insulin secretion in βICKO islets induced ex vivo (24h o/n 1micrmoM Tamoxifen or Etanhol)(n=3; vehicle p=0.0001; Tx Treated p=0.0001; vehicle High glucose vs tx treated high glucose p=0.0172(t-test) , mean±s.d.) **B.** Glucose stimulated insulin secretion in BBS4$^{/-}$ and BBS4$^{+/+}$islets (n=4, control p=0.0028; WT high glucose vs HOM high glucose p=0.0019 (ttests) , mean±s.d.). **C.** Immunoflorescent staining for mTomato (red) and mGFP (green) of brain cryosections from Pdx1^{CreER} mT/mG Cre$^{+/-}$ mice induced with tamoxifen. **D.** Immunoflorescent staining for mTomato (red) and mGFP (green) of pancreas cryosections from Pdx^{CreER} mT/mG Cre$^{+/-}$ mice induced with tamoxifen. **E.** Immunoflorescent staining for mTomato (red) and mGFP (green) and Gluagon (cyan) of pancreas cryosections from Pdx1 mT/mG Cre$^{+/-}$ mice induced with tamoxifen.

Suppl. Fig. 4

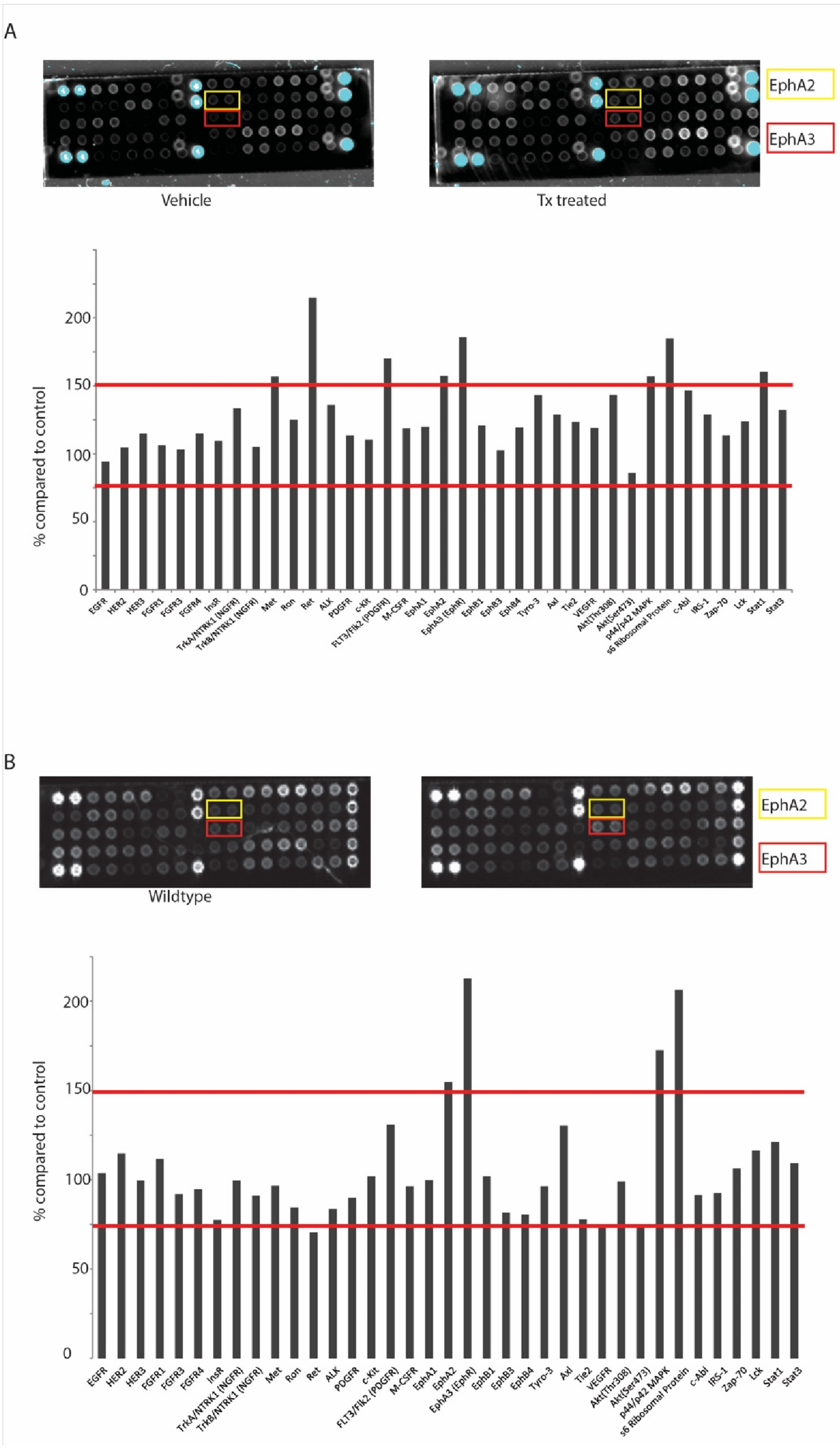

Supplemental Figure 4: RTK PathScan of BICKO and Bbs4$^{-/-}$ islets.

A. Digital images of single wells incubated with vehicle (left) and Tx treated (right) islet lysates from BICKO mice. Spots with antibodies against pEphA2 (all potential phosphoTyr sites) are highlighted in yellow, pEphA3 (all potential phosphoTyr sites) in red with quantifactions, red line place at 75 and 125%. **B.** Digital images of single wells incubated with wildtype (left) and Bbs4$^{-/-}$ (right) islet lysates. Spots with antibodies against pEphA2 (all potential phosphoTyr sites) are highlighted in yellow, pEphA3 (all potential phosphoTyr sites) in red .

Suppl. Fig. 5

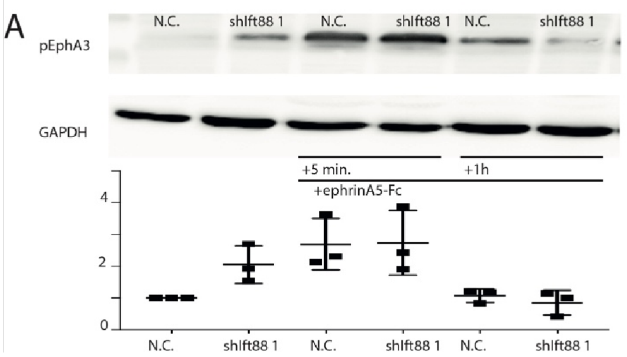

Supplemental Figure 5: Glucose stimulated insulin secretion is linked to EphA dephosphorylation defects.

A. Immunoblotting of phosphorylated EphA3 (Tyr779) and GAPDH in one clone of MIN6m9 cells expressing shIft88 (shift88 1) and scrambled RNA (N.C.) treated with ephrinA5-Fc for 1 hour and 5 minutes and quantification, mean$\pm$s.d..

Supplemental Table 1. Baseline characteristics of pancreas biobank cohort

	Overall
N	19
Sex = male (%)	15 (78.9)
Age (mean (sd))	66.43 (9.31)
BMI (mean (sd))	26.79 (4.85)
HBA1C (mean (sd))	6.16 (1.21)
glycemic (%)	
Normal glucose tolerance (%)	3 (15.8)
Prediabetes (%)	8 (42.1)
Diabetes (%)	8 (42.1)

Original blots

Fig 2A

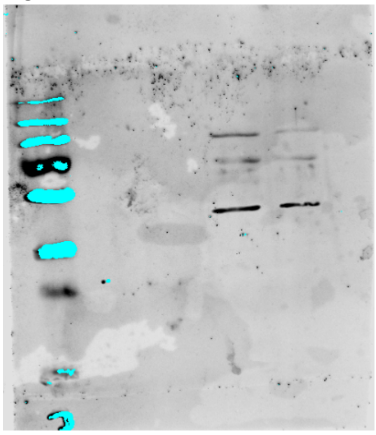

Fig 2B pEphA3

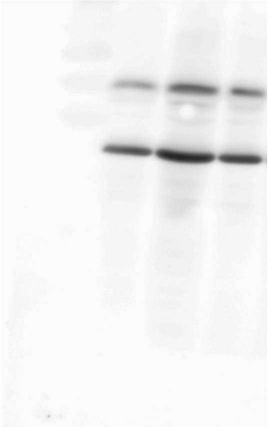

Fig 2B EphA3

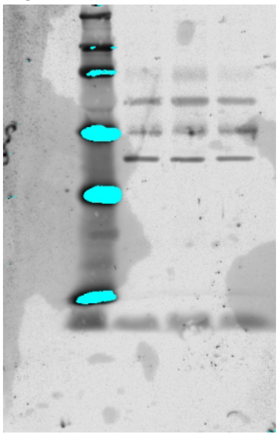

Fig 2B ephrinB1

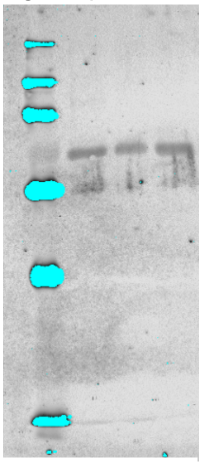

Fig 2C pERK islets

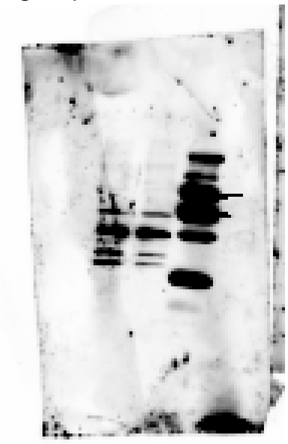

Fig 2C ERK islets

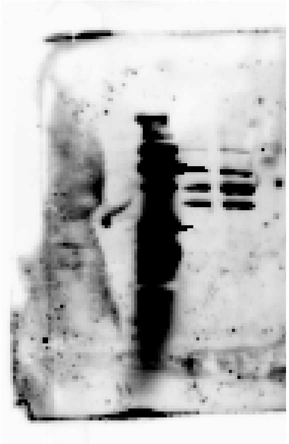

Fig 2C pERK MIN6

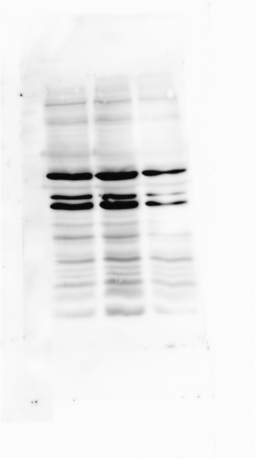

Fig 2C ERK MIN6

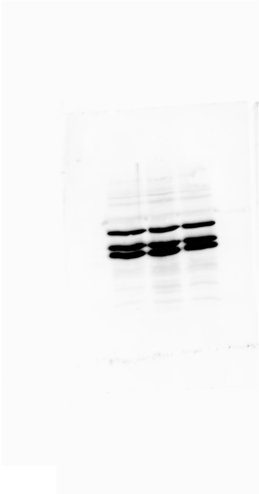

Fig 2C pAKT

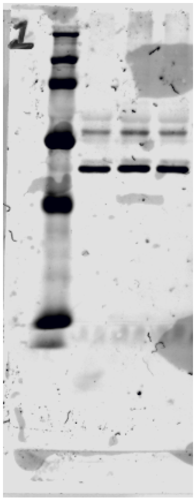

Fig 2C panAKT

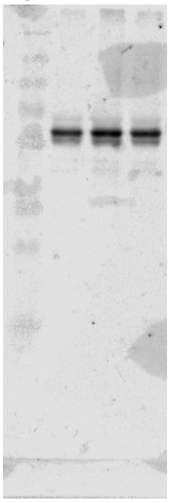

Fig 2C pPI3K

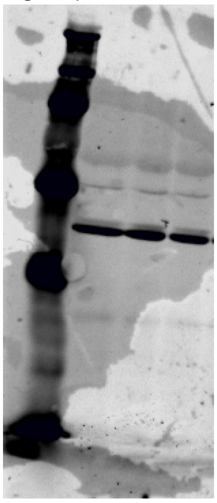

Fig 2C panPI3K

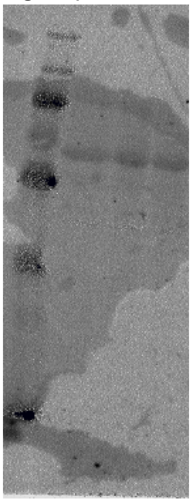

Fig 3D

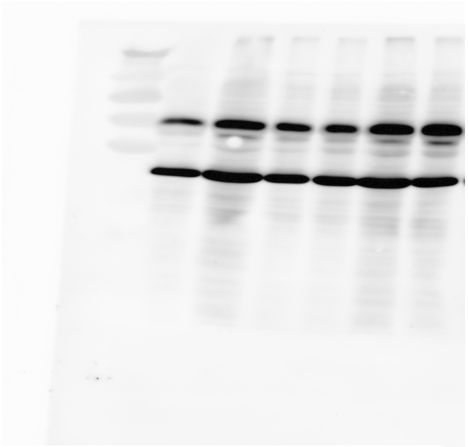

Fig 3E

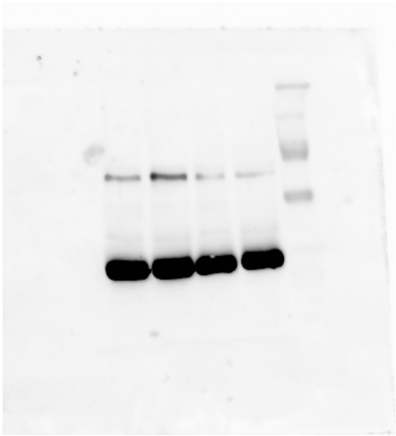

Fig 4A pEphA3

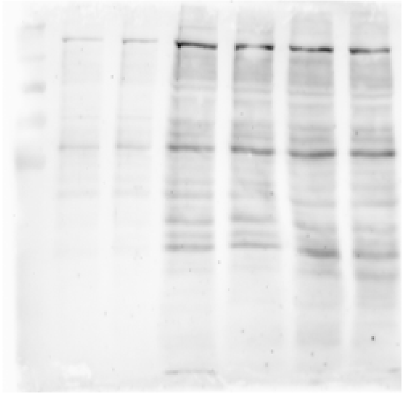

Fig 4A EphA3

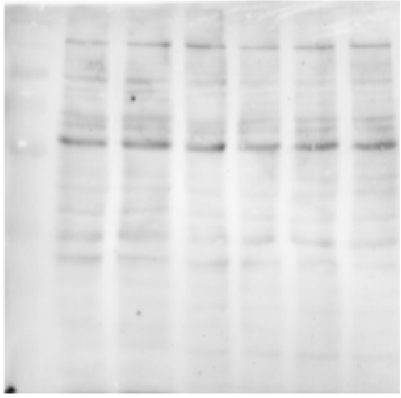

Fig 4A Actin

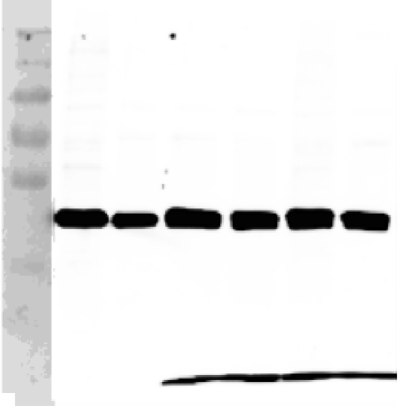

Fig 4B pEphA3

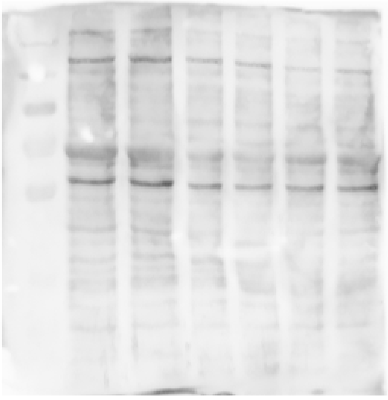

Fig 4B Actin

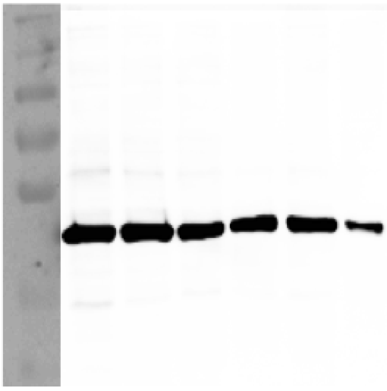

Fig 4C pEphA3

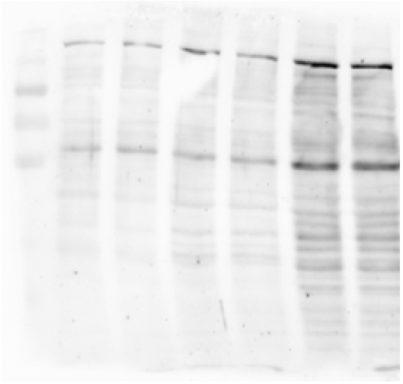

Fig 4C EphA3

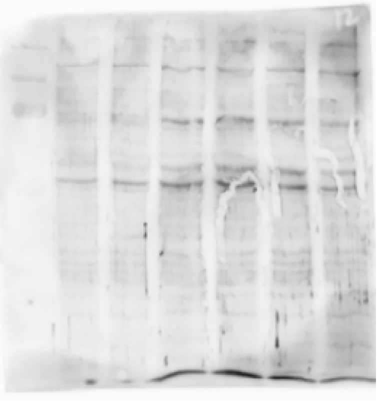

Fig 4C Actin

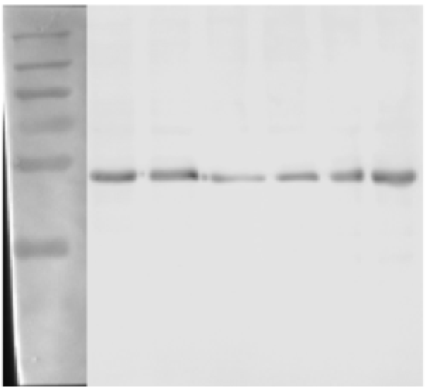

Fig 5A panPtp inhibitor

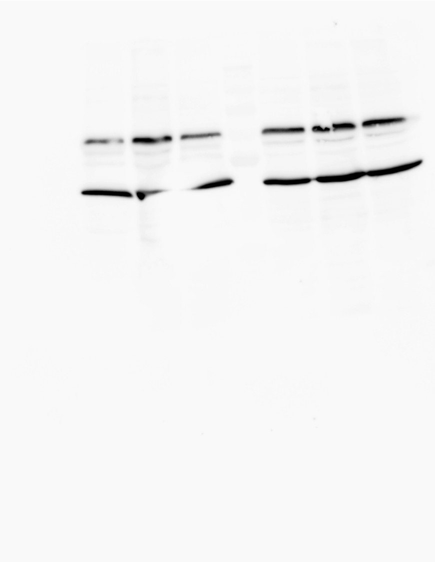

Fig 5A Ptp1b inhibitor

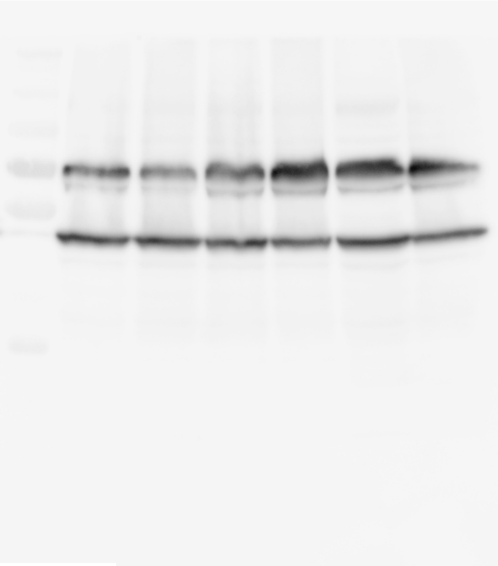

Fig 6A F/G actin

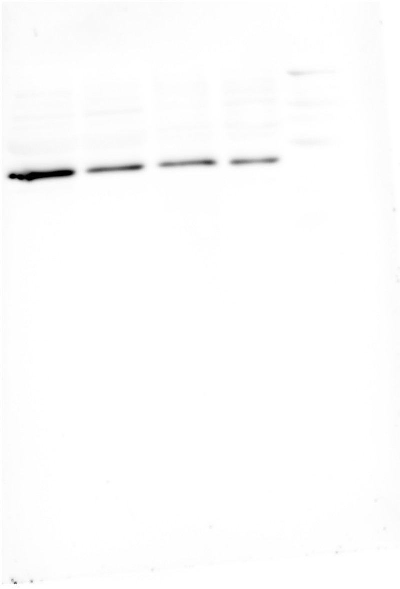

Fig 6D b-catenin

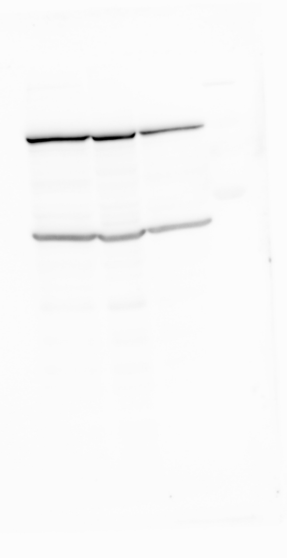

Fig 6D snail

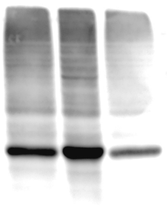

Fig 6D Slug

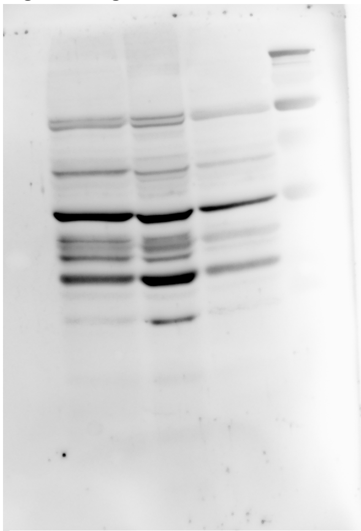

Fig 6D Vimentin

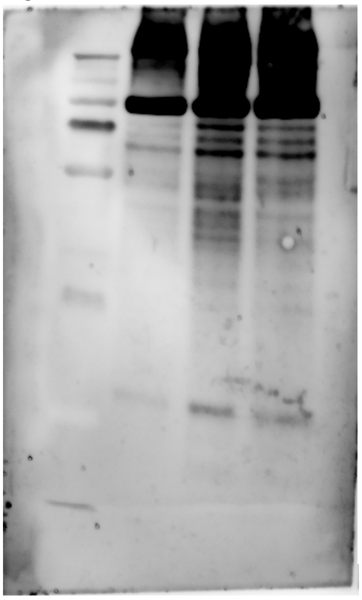

Fig 6D HSP 90

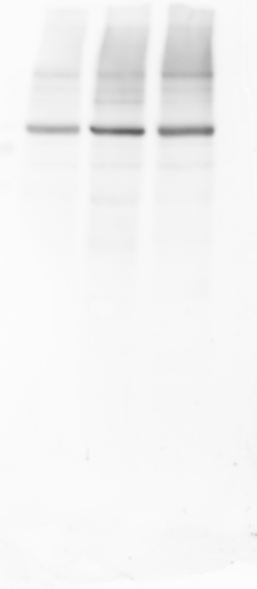

Fig 6D E-cadherin

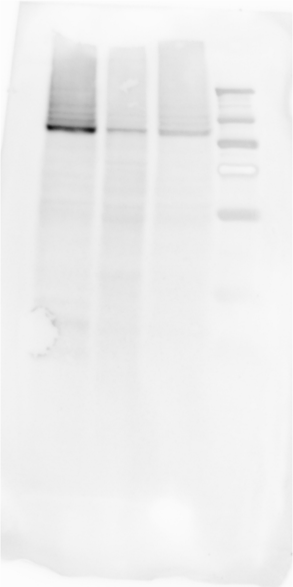

Fig 6E GTP-Rac1

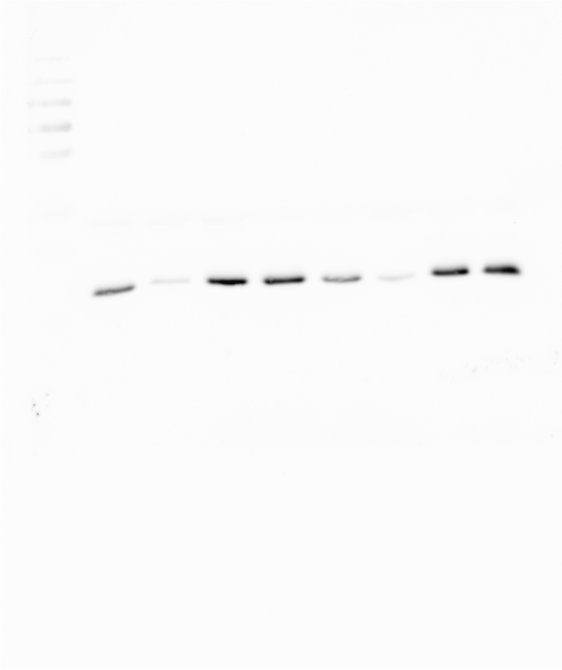

Fig 6E Rac1, actin

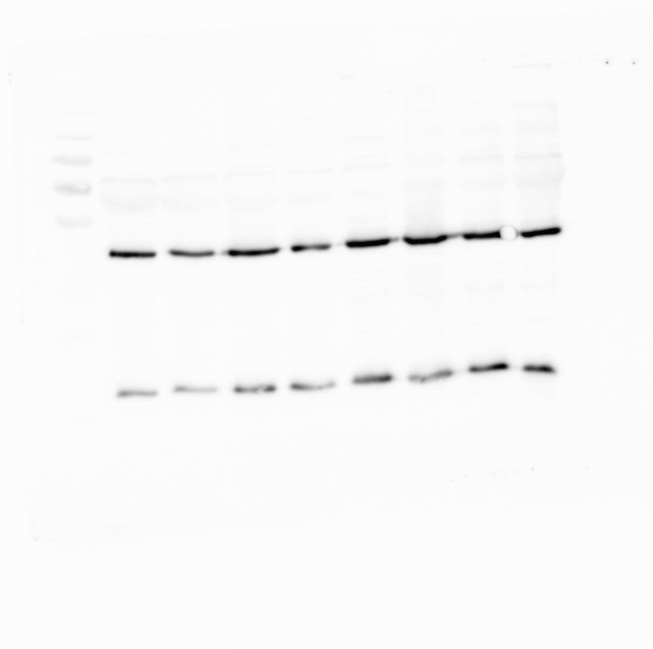

Fig 7A

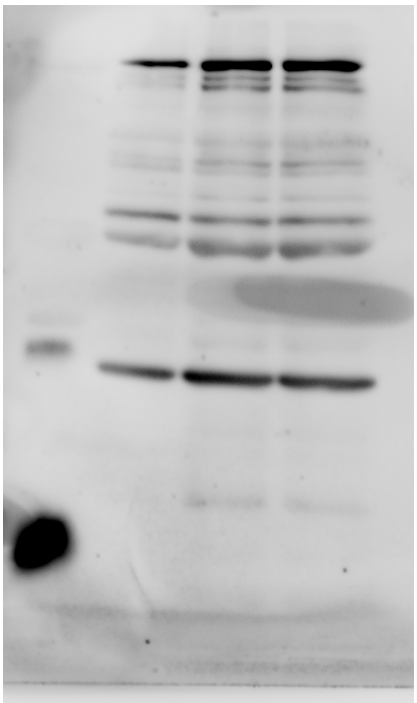

Fig 8B R229 R232 pEPHA3

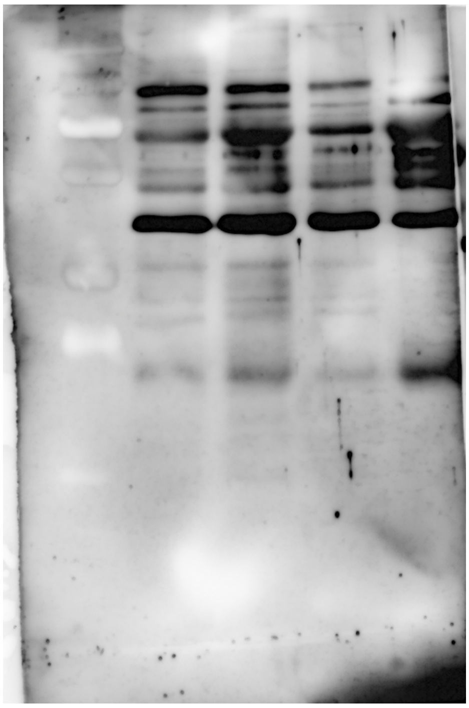

Fig 8B R229 R232 IFT88

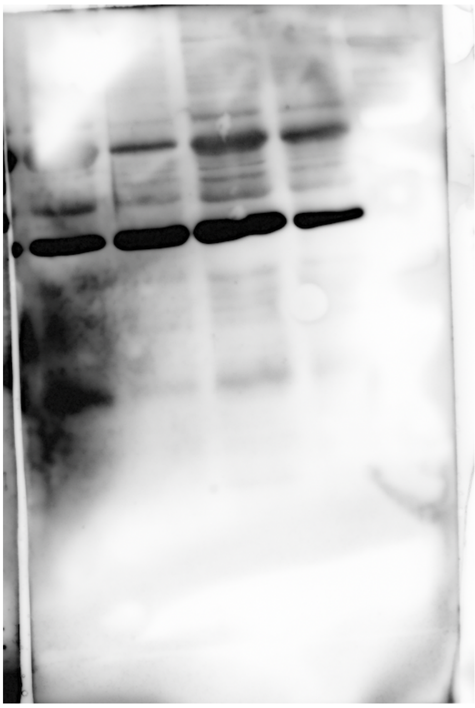

Fig 8B R233 pEPHA3

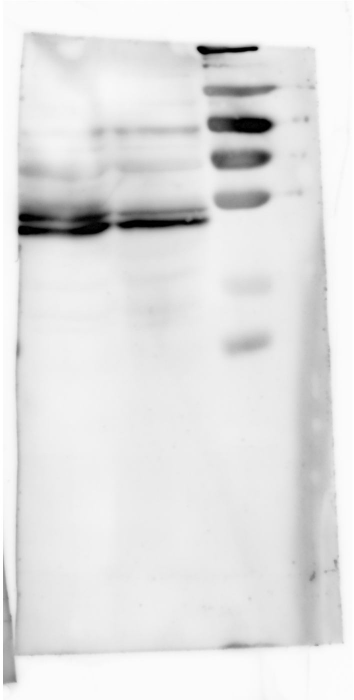

Fig 8B R233 IFT88

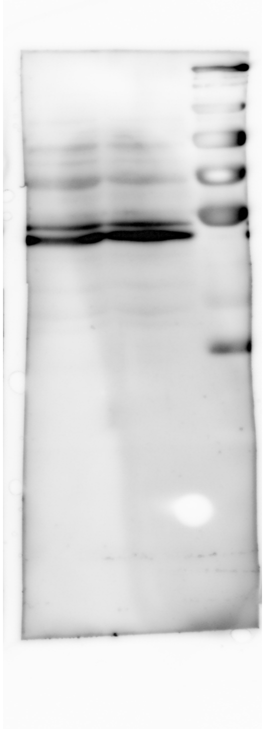

Fig 8B R230

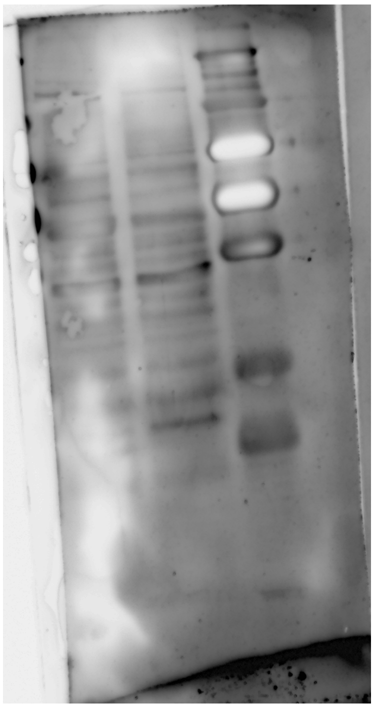

Fig 8D R268, R262, R263, R259

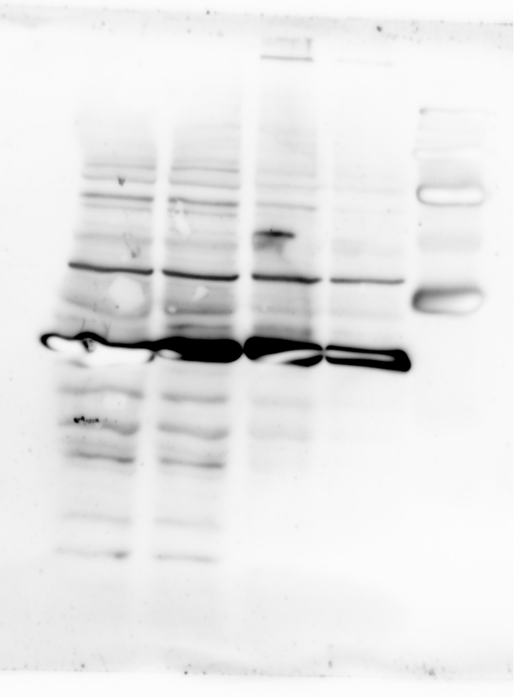

Fig 8D R259, R271

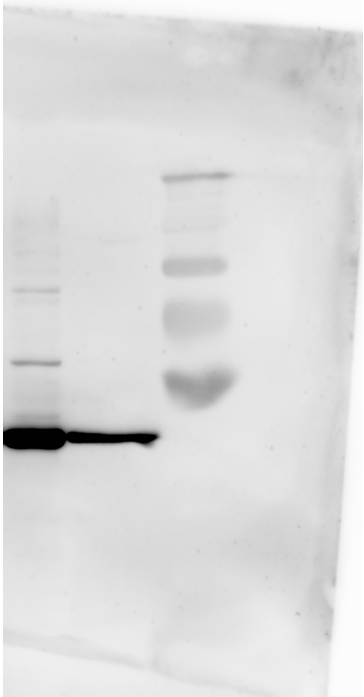

Supplement: Supplementary file 1 — Supplementary Information [file 41467_2019_12953_MOESM1_ESM.pdf]
