# Supplementary material for: Glucose homeostasis is regulated by pancreatic β-cell cilia via endosomal EphA-processing
Source: Nat Commun. 2019 Dec 12;10:5686. doi: 10.1038/s41467-019-12953-5 (PMC6908661; doi:10.1038/s41467-019-12953-5)
Supplement: Supplementary file 3 — Reporting Summary [file 41467_2019_12953_MOESM3_ESM.pdf]

nature research

Corresponding author(s): Jantje Gerdes

Last updated by author(s): 2019-10-4

Reporting Summary

Nature Research wishes to improve the reproducibility of the work that we publish. This form provides structure for consistency and transparency in reporting. For further information on Nature Research policies, see Authors & Referees and the Editorial Policy Checklist.

Statistics

For all statistical analyses, confirm that the following items are present in the figure legend, table legend, main text, or Methods section.

n/a	Confirmed	
☐	☒	The exact sample size (*n*) for each experimental group/condition, given as a discrete number and unit of measurement
☐	☒	A statement on whether measurements were taken from distinct samples or whether the same sample was measured repeatedly
☐	☒	The statistical test(s) used AND whether they are one- or two-sided<br>*Only common tests should be described solely by name; describe more complex techniques in the Methods section.*
☒	☐	A description of all covariates tested
☐	☒	A description of any assumptions or corrections, such as tests of normality and adjustment for multiple comparisons
☐	☒	A full description of the statistical parameters including central tendency (e.g. means) or other basic estimates (e.g. regression coefficient) AND variation (e.g. standard deviation) or associated estimates of uncertainty (e.g. confidence intervals)
☐	☒	For null hypothesis testing, the test statistic (e.g. *F*, *t*, *r*) with confidence intervals, effect sizes, degrees of freedom and *P* value noted<br>*Give P values as exact values whenever suitable.*
☒	☐	For Bayesian analysis, information on the choice of priors and Markov chain Monte Carlo settings
☒	☐	For hierarchical and complex designs, identification of the appropriate level for tests and full reporting of outcomes
☐	☒	Estimates of effect sizes (e.g. Cohen's *d*, Pearson's *r*), indicating how they were calculated

Our web collection on statistics for biologists contains articles on many of the points above.

Software and code

Policy information about availability of computer code

Data collection	No specific software was used
Data analysis	SigmaPlot 12.0, Excel, GraphPad outlier analysis

For manuscripts utilizing custom algorithms or software that are central to the research but not yet described in published literature, software must be made available to editors/reviewers. We strongly encourage code deposition in a community repository (e.g. GitHub). See the Nature Research guidelines for submitting code & software for further information.

Data

Policy information about availability of data

All manuscripts must include a data availability statement. This statement should provide the following information, where applicable:

- Accession codes, unique identifiers, or web links for publicly available datasets
- A list of figures that have associated raw data
- A description of any restrictions on data availability

Data is available under Fileshare (as stated in manuscript)

Field-specific reporting

Please select the one below that is the best fit for your research. If you are not sure, read the appropriate sections before making your selection.

☒ Life sciences ☐ Behavioural & social sciences ☐ Ecological, evolutionary & environmental sciences

For a reference copy of the document with all sections, see nature.com/documents/nr-reporting-summary-flat.pdf

Life sciences study design

All studies must disclose on these points even when the disclosure is negative.

Sample size	Sample size for animal testing was N=6 as approved by the national authorities and verified by power analysis. In other experiments, we performed at least N=3 independent, biological experiments and measured in technical duplicates.
Data exclusions	We performed Grubbs' outlier analysis (GraphPad) and excluded statistical outliers.
Replication	S.a., we performed at least three independent biological experiments. The animal cohorts were followed over time and tested at several time points.
Randomization	Due to the relatively small cohort, we did not perform randomization in our animal experiments.
Blinding	In the animal experiments, the experimenter was blinded to the previous treatment (Oil/ Tx) or genotype (BICKO or floxed Ift88) of the animals.

Reporting for specific materials, systems and methods

We require information from authors about some types of materials, experimental systems and methods used in many studies. Here, indicate whether each material, system or method listed is relevant to your study. If you are not sure if a list item applies to your research, read the appropriate section before selecting a response.

Materials & experimental systems

n/a	Involved in the study	
☐	☒	Antibodies
☐	☒	Eukaryotic cell lines
☒	☐	Palaeontology
☐	☒	Animals and other organisms
☐	☒	Human research participants
☒	☐	Clinical data

Methods

n/a	Involved in the study	
☒	☐	ChIP-seq
☒	☐	Flow cytometry
☒	☐	MRI-based neuroimaging

Antibodies

Antibodies used	All antibodies used are described incl. cat # and manufacturer in the Materials and Methods section.
Validation	S.a.

Eukaryotic cell lines

Policy information about cell lines

Cell line source(s)	MIN6m9
Authentication	no authentication was carried out, cells were shipped directly from S. seino's lab, the originator of this cell line.
Mycoplasma contamination	Min6m9 cells are tested regularly for mycoplasma contamination and have been tested negative for the past year.
Commonly misidentified lines (See ICLAC register)	None used

Animals and other organisms

Policy information about studies involving animals; ARRIVE guidelines recommended for reporting animal research

Laboratory animals	We used conditionally targeted alleles placing the floxed Ift88 allele (B. Yoder's lab) under the control of the Pdx1- promoter (M. Gannon's lab). The background was mixed C57B6/J and C3H3. We used male animals. For number and age please refer to Manuscript.
Wild animals	Study did not involve wild animals.
Field-collected samples	No field-collected samples were used in this study.
Ethics oversight	Ethics oversight over animal experiments is carried out by the Regierung of Oberbayern.

Note that full information on the approval of the study protocol must also be provided in the manuscript.

Human research participants

Policy information about studies involving human research participants

Population characteristics	The PPP biobank participants were recruited at the University Clinic Tübingen (N=19). Please refer to Supplemental Table for population characterstics. The pancreatic islets were made available by the IsletCore at the University of Alberta, Edmonton, Canada. Characteristics of individual donors are described in the manuscript.
Recruitment	S.a. Informed consent was given by study pariticpants or relatives respectively.
Ethics oversight	Human Ethics Commission in Munich (Az 557/16S); Ethics Commission of the Medical Faculty of the University of Tübingen (#697/2011BO1)

Note that full information on the approval of the study protocol must also be provided in the manuscript.
